# Preliminary Investigations on the Pyrometallurgical Recycling of a TiMn_2_–Based Hydrogen Storage Alloy

**DOI:** 10.3390/ma19091804

**Published:** 2026-04-28

**Authors:** Jan Krusenbaum, Ajithkumar Selvan, Bernd Friedrich

**Affiliations:** IME Metallurgical Processing and Metal Recycling, RWTH Aachen University, 52072 Aachen, Germany; aselvan@metallurgie.rwth-aachen.de (A.S.); bfriedrich@metallurgie.rwth-aachen.de (B.F.)

**Keywords:** hydrogen storage alloy, TiMn_2_-based intermetallic, gas impurities, pyrometallurgical recycling, melt–refractory interactions

## Abstract

Hydralloy^®^ C5, an intermetallic TiMn_2_-based alloy, has been manufactured industrially (GfE, Nuremberg) for decades and is used on a large scale for hydrogen storage. During use, the alloy is stored in gas-tight and pressure-resistant storage containers. At the end of service, the alloy is a fine powder with pyrophoric character (Ti- and Zr- content). This significantly hinders the safe extraction from the containers and subsequent recycling of the alloy due to unavoidable reactions with ambient air. The major concern on passivation and maximum permissible content with O/N must be clarified for safe handling in ambient air as well as regarding the pyrometallurgical recycling. Considering this, and in preparation for the opening of real large-scale storage containers, end-of-life Hydralloy C5 was synthesized with two different levels of O (~0.15 and ~1 wt.%) and N (~0.04 and ~8 wt.%) contamination. Vacuum induction melting (VIM) and cold crucible arc melting (CCAM) were chosen as potentially suitable for recycling. The preliminary remelting trials from both aggregates ascertained that the recovery of metal content is not feasible with heavily O/N-contaminated alloys. It is concluded that extreme caution should be taken to minimize contamination when extracting the powdered alloy from the storage containers. Hydralloy C5 with moderate gas impurities (~0.15 wt.% O and ~0.04 wt.% N) can be remelted, on the other hand, in both VIM and CCAM. Contact between molten Hydralloy C5 with selected refractories (Al_2_O_3_-TiO_2_ and CaO-stabilized ZrO_2_) in the VIM leads to the formation of a multi-layered transition zone dominated by Ti and Zr. While the Al_2_O_3_ in the titanium aluminate is infiltrated and reduced by Ti and Zr, the crucible wall made of CaO-stabilized ZrO_2_ remains intact. Despite low gas contents, significant losses in melt yield are recognized due to crucible wall deposits from the formation of non-metallic inclusions during VIM. Against this background, the use of fluxes is being considered for future melts in addition to the use of deoxidants.

## 1. Introduction

**Figure 1:**
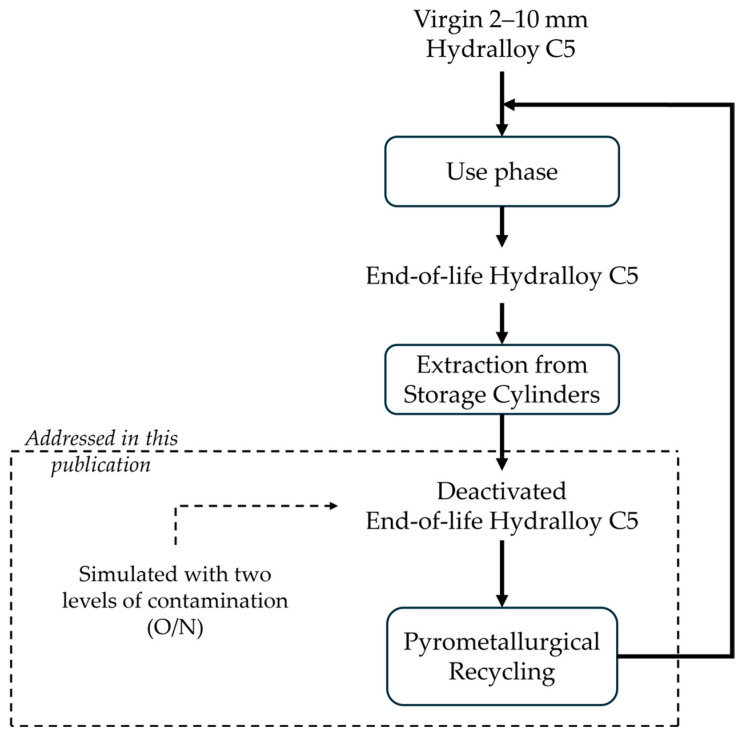
Life cycle of Hydralloy C5 and focus of this paper.

Hydralloy C5 is a hydrogen storage alloy (HSA) that was patented in the 1980s and has been commercially available for some time [1]. This alloy (trademark HYDRALLOY^®^ C5; A = Ti, Zr; B = Mn, Ni, V, Fe, Cr) is a TiMn_2_-based (AB_2_) alloy [2]. For operation it is stored in gas-impermeable steel containers. The design of such a hydrogen storage cylinder is, for example, described in a patent specification from 1987 [3] or can be seen in Figures 1 and 10 of Lázár et al. [4]. During use the alloy undergoes recurring absorption and desorption cycles. This leads over time to the pulverization of the alloy (called decrepitation) down to particles sizes < 100 µm [5] (p. 117).

There is no recorded information on the service duration of alloy-filled storage containers. However, the question of their possible destination after their initial area of operation exists. Secondary utilization could be considered but would not resolve the inquiry of the final destination. Landfilling is not an option as a final solution in the case of this alloy, as compounds of some alloy constituents pose serious health and environmental risks. For instance, MnO_2_ (>80 µg*m^−3^) in water and V_2_O_5_ dust (>20 µg*m^−3^) are acutely toxic for humans by inhalation/consumption [6,7]. In addition to the ecological reasons for establishing a recycling process, there are also economic and geostrategic arguments for metal recovery. On the one hand, the European Union is dependent on imports for a wide range of raw materials (known as critical raw materials (CRMs)) (see Section 1.2). On the other hand, a recent life cycle assessment study [8] shows that the development of a recycling process has the potential to lead to significant savings in CO_2_ emissions (see Section 1.3).

In order to recycle the alloy to a reusable condition, it must first be removed from the air- and gas-tight containers. However, due to its particle size range of <100 µm, the alloy has an extremely large surface area. This, combined with its oxygen affinity [3], will lead to a partial contamination of the alloy powder (depending on the surrounding medium), which must be removed during subsequent recycling to restore the hydrogen storage capability. A certain degree of contamination is unavoidable (as a recycling process chain completely under protective gas is extremely unrealistic for cost reasons) and even necessary, as it leads to passivation and thus makes the otherwise pyrophoric alloy powder manageable in ambient air. This process is referred to as deactivation and is used throughout this publication.

It is unclear how high the contamination must be in order to obtain a manageable product and, at the same time, how low it must be in order to make pyrometallurgical recycling possible. Answers to these questions are necessary, also with regard to the design of safe opening procedures for real, large-scale storage containers. Until these issues have been addressed, opening a real storage container would be too dangerous. This is why this publication does not include a direct comparison with the real contaminated end-of-life Hydralloy C5. Against this background, deactivated end-of-life Hydralloy C5 is first produced synthetically with two different levels of contamination (O/N). The two methods used for this also reflect two different opening methods in terms of the availability of the deactivation medium. Because it is the cheapest medium, ambient air is chosen as the deactivation medium. After synthesis, the material is comprehensively characterized before it is used afterwards in the preliminary remelting trials. The intended life cycle of Hydralloy C5 and the approach pursued in this publication are shown in Figure 1 as a flowchart.

The aspects covered in this publication are listed below.

- Simulation of deactivated end-of-life Hydralloy C5 by synthesizing with varying levels of oxygen and nitrogen contamination;
- Characterization of synthetically deactivated end-of-life as well as virgin Hydralloy C5 (ICP-OES, XRD, hot gas extraction, microscopic and SEM-EDX analysis);
- Determination of Hydralloy C5’s basic properties in the molten state (melting/solidification temperature and vapor pressure) experimentally and by simulation;
- Preliminary remelting trials of the synthetically deactivated end-of-life materials via VIM and CCAM (including investigation of the interaction between liquid Hydralloy C5 and selected refractory materials).

Initial research indicates that the field of hydrogen storage alloy recycling is heavily concentrated on AB_5_ alloys (mainly LaNi_5_) to date. The most likely reason for this is the use of LaNi_5_-based alloys in the negative electrodes of widely used nickel metal hydride batteries (NiMHBs). Recovery of rare earth elements (REEs) (Ce, La, Pr and Nd) were mentioned in the recycling of NiMHBs via pyro- and hydrometallurgical methods by review publications like [9,10,11,12,13]. To the best of our knowledge, there is no research on the recycling of spent-TiMn_2_-based alloys from storage vessels yet.

Since the TiMn_2_-based Hydralloy C5 is a fairly niche product, the next subchapter (Section 1.1) will be used to provide a few more brief explanations about this alloy and its primary production. The latter is important in order to better assess the criticality and dependency of raw materials, which will then be discussed in Section 1.2. Section 1.3 then takes a closer look at the ecological benefits of developing a recycling process.

### 1.1. Hydralloy C5—A Short Overview

The alloy is characterized by a maximum storage capacity of ~1.8 wt.% at 20 °C [14]. This in combination with the possibility of carrying out hydrogen loading and unloading between −20 °C and 100 °C [15] makes it a marketable product. According to GfE’s own website, the alloy is used for energy supply in submarines, excursion boats, forklift trucks, and special vehicles [16]. Ref. [15] also mentions use in non-mechanical hydrogen compressors.

The primary manufacturing process of the alloy is described by [1,17] as a two-step vacuum induction melting (VIM). First, ferrovanadium master alloy is produced using aluminothermy. In the first vacuum induction melting step, a MnVFe intermediate alloy is melted at 1400 °C with the addition of electrolyte manganese. This is broken up and melted in a second stage at approximately 1300 °C with the addition of the refractory metals titanium and zirconium (sponge form), as well as other alloy components if required. Since the hydrogen storage capacity decreases significantly at too high oxygen contents [18], cerium mischmetal is added for deoxidation shortly before casting. Without explicit deoxidation, the oxygen content would be approx. 0.4–0.6 wt.%, with ferrovanadium containing up to 0.9 wt.% and thus contributing the most to the mentioned oxygen range [1]. In addition to oxygen, there are other impurities that have a negative impact on hydrogen storage capabilities. Of relevance here is not only the maximum storage capacity, but also the required plateau pressures. Morita et al. [19] investigated a Ti_0.9_Zr_0.1_Mn_1.4_Cr_0.4_V_0.2_ alloy and noted that the plateau pressure increases significantly with elements such as B, (O), S, Se and C, i.e., the hydrogen pressure required for storage. This, together with maintaining the chemical composition necessary for the formation of the phase(s) required for hydrogen storage capability (in the case of Hydralloy C5, this is C14 Laves), is of essential importance to consider during pyrometallurgical processing.

The main raw materials used in primary production are therefore iron, aluminum, and vanadium oxide in the case of in-house production, or FeV in the case of direct procurement. However, the majority of the raw materials used are electrolytic manganese and titanium sponge (and zirconium sponge). Cerium mischmetal is used as deoxidizer, which after [20] (p. 32) consists mainly out of cerium (50%), lanthanum (25%) and neodymium (15%).

### 1.2. Criticality of Raw Materials and Supply Risks

Every three years since 2011, the EU has published a list of raw materials classified as critical. The most recent list is from 2023. With 34 entries, it contains significantly more than the 14 from 10–15 years ago. Raw materials from this list are referred to as critical raw materials (CRMs). If they are also essential for the EU’s strategic interests in terms of security and future technologies, they are also classified as strategic raw materials (SRMs). Table 1 shows the inputs required for the primary production of Hydralloy C5 and their classification according to the EU. In addition to criticality, the EU import reliance (IR) for primary and refined products is also listed. Ref. [21] defines IR as the “ratio between net imports and the sum of net imports and domestic production.” The table shows that the vast majority of input materials are classified as critical or critical and strategic.

The above explanations show that multiple input materials must be used for the primary production of hydrogen storage alloys, for which there is a high degree of import dependency. In times of increasing geopolitical shifts and uncertainties, it makes sense to recycle disused goods located on one’s own soil.

### 1.3. Ecological Impact of Hydralloy C5

In addition to the geopolitical perspective, there are also business-economic and ecological perspectives that support the recycling of the alloy. These are represented by a life cycle assessment (LCA). Creating an LCA requires a lot of background information and special software with extensive databases. Against this background, it is gratifying that a very recent (published in 2025) LCA is available specifically for Hydralloy C5. The LCA mentioned refers to the publication of Puszkiel et al. [8]. The modeling is carried out with 1.25 t of Hydralloy C5, replicating industrial manufacturing scale. The model is executed as a cradle-to-operation approach (system boundaries) for the Hydralloy composition of Ti_0.95_Zr_0.05_Mn_0.730_V_0.225_Fe_0.045_. This means that it includes the raw material extraction, production, transport and use phase.

Two scenarios are directly compared: conventional production from primary raw materials and the manufacture of the alloy from secondary materials (“metal scrap (post-use)”). Figure 2 from [8] shows the comparison between the scenarios in terms of global warming potential (GWP). In addition to the two scenarios, a middle variant is also shown, in which primary and secondary input materials are mixed. The resulting GWP as kg CO_2_-eq per kg of Hydralloy C5 alloy produced are 28.6, 20, and 5.8 for the primary, mixed, and secondary scenarios, respectively. This significant decrease is mainly caused by the extreme CO_2_ emissions related to the raw material extraction and processing. These raw-material-related emissions account for 82.3% in the case of the primary production scenario with titanium production comprising approx. 50% of this.

The life cycle evaluation case study of Hydralloy C5 by Puszkiel et al. [8] demonstrates that the production of the alloy from secondary resources has great potential in terms of reducing CO_2_-eq emissions.

The last two subchapters made clear that recycling end-of-life Hydralloy C5 is a sensible thing to do. Since, to the best of the author’s knowledge, no research on recycling this alloy has been conducted to date, this topic will be addressed here and in further publications.

## 2. Preparatory Work

### 2.1. Synthesis of Deactivated End-of-Life Hydralloy C5

Deactivated end-of-life Hydralloy C5 with low contamination and one with high contamination is synthesized.

The high level of contamination is achieved using the following process (corresponds to the fastest, simplest, and cheapest deactivation and extraction method—it mirrors the abrupt opening of the real storage containers): The 2–10 mm alloy is charged into a Sieverts device for measuring H_2_ storage capacity. After closing the apparatus, the alloy is cycled several times (charged and discharged with H_2_) until it is pulverized. Once this is complete, the chamber lid is opened abruptly, exposing it to surrounding atmosphere. Following this the material is tipped into a collection container.

**Figure 3:**
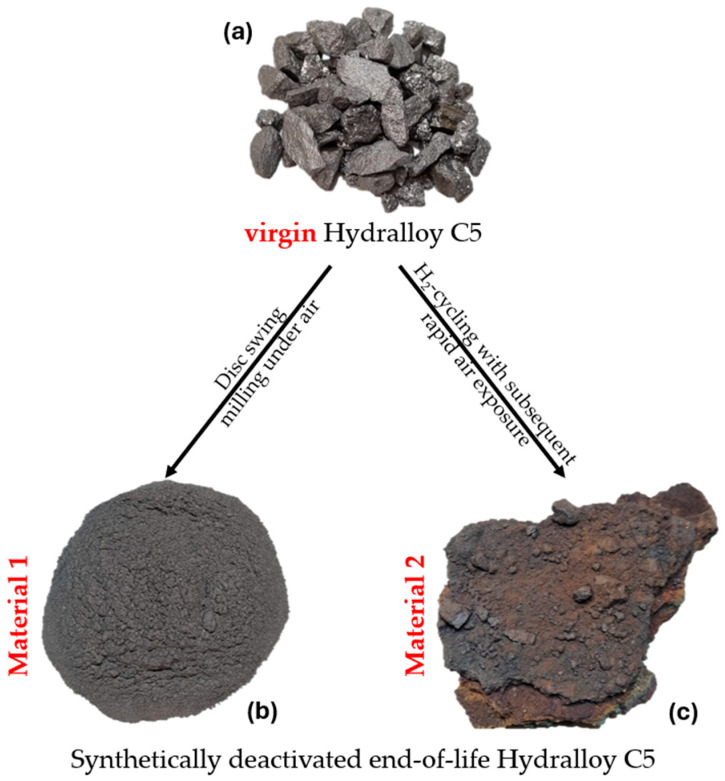
Virgin Hydralloy C5 2–10 mm as shipped by GfE (**a**); 2–10 mm virgin material ground for 20 s in air (Material 1) (**b**); cycled multiple times with hydrogen and subsequently rapidly exposed to ambient air atmosphere (Material 2) (**c**).

For the low contamination, the brittleness of the intermetallic alloy is exploited by grinding the 2–10 mm granules with a disc swing mill (TS.250, SIEBTECHNIK GmbH, Mülheim an der Ruhr, Germany) for a very short time (20 s) sealed under air. Figure 3 shows virgin 2–10 mm Hydralloy C5, i.e., the starting material (a), as well as the materials generated by grinding (from now on called Material 1) (b) and H_2_-cycling in combination with rapid ambient air exposure (from now on called Material 2) (c).

The materials seen in Figure 3 are systematically characterized using ICP-OES, X-ray diffraction (XRD), hot gas/carrier gas extraction, light microscopy (VH-S30K, KEYENCE DEUTSCHLAND GmbH, Frankfurt am Main, Germany) and SEM-EDX (GeminiSEM 500, Carl Zeiss AG, Oberkochen, Germany). To analyze via XRD, samples are ground and sieved to <90 μm and then handed over to IFK RWTH Aachen University. ICP-OES and hot gas extraction were performed at IME RWTH Aachen University, unless otherwise noted. While light microscopy was performed at the IME, all SEM-EDX images shown in this publication were taken at the GHI RWTH Aachen University.

#### 2.1.1. Composition and Gas Analysis

**Figure 4:**
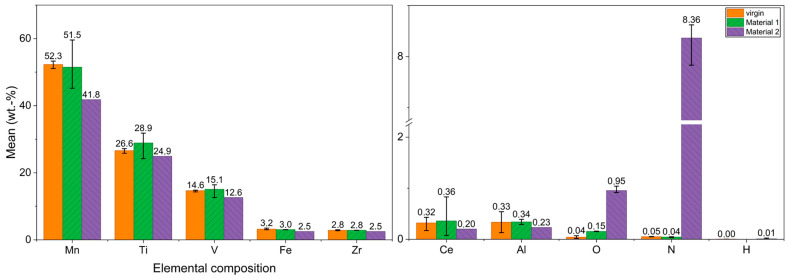
Composition (all values except for H of Material 2 and virgin Hydralloy C5 were determined at the IME of RWTH Aachen University; H was determined by Fraunhofer IFAM in Dresden) of virgin Hydralloy as well as Material 1 and Material 2 (the ends of the error bars correspond to the measured minimum and maximum values).

The results of the ICP-OES and gas analysis are shown in Figure 4 (measured as seen in Figure 3) for the virgin alloy as well as Material 1 and Material 2. The five main alloying elements Mn, Ti, V, Fe, and Zr are listed, as well as the minor elements plus O and N. H was also analyzed. Hydrogen amounts to 0.002 and 0.008 wt.% for virgin and Material 2, respectively. There are virtually no traces left from the hydrogen loading in the case of Material 2. Due to these extremely low contents and since Material 1 originates directly from the virgin alloy, a H measurement was not performed for Material 1. Mn makes up the largest proportion of the virgin alloy, accounting for around 50% of the total content, followed by Ti and V in a low double-digit content range. Fe and Zr both amount to approx. 3 wt.%. The minor elements Al and Ce originate from the manufacturing process. Al comes from the aluminothermic production of FeV and the Ce from the cerium mischmetal used for deoxidation. Converted into atomic percent, the chemical composition of the virgin Hydralloy C5 yields the intermetallic AB_2_ stoichiometry. The B elements Mn, V, and Fe account for 68.85 at. %, while the A elements Ti and Zr account for 31.15 at.%. A look at the main alloy element composition of Material 2 shows that all have lost content. This is due to enrichment with oxygen and nitrogen. The sudden contact between the powdered alloy and the ambient atmosphere led to severe oxidation and nitriding. It is particularly noteworthy that significantly more material was nitrided than oxidized. Compared to the virgin alloy, the O and N values rose from 0.04 to 0.95 wt.% and from 0.05 to 8.36 wt.%, respectively. In contrast, there was virtually no change in the composition of Material 1 (ground virgin alloy). The rather large error bars are most likely due to partial inhomogeneities in the powder. These fluctuations occur even though the powder was mixed for approx. 5 min beforehand (MUK mixer, FLUXANA GmbH & Co. KG, Bedburg-Hau, Germany). In contrast to Material 2, the O content only increased from 0.04 to 0.15 wt.%. The N content is virtually unchanged at 0.04 wt.%.

**Figure 5:**
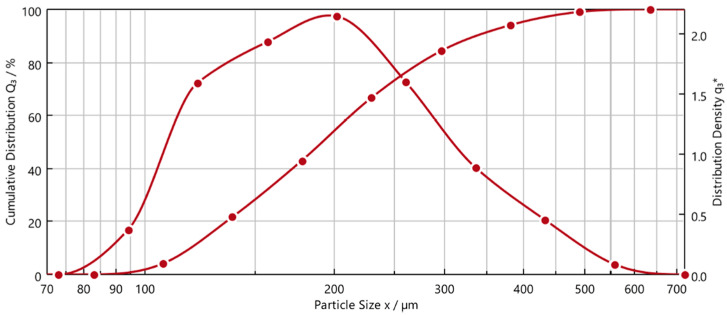
Particle size distribution of Material 1.

Figure 5 depicts the particle size distribution of Material 1 (QICPIC RODOS/L VIBRI/L, Sympatec GmbH, Clausthal-Zellerfeld, Germany). The particle size spectrum ranges from approx. 75 to 650 µm with a d_50_ of 190 µm. The shape descriptor (measure of sphericity, where 1 is a perfect sphere and 0 describes an elongated, flat “line-like” particle) is between 0.7 and 0.8 across the entire spectrum.

#### 2.1.2. X-Ray Diffraction

**Figure 6:**
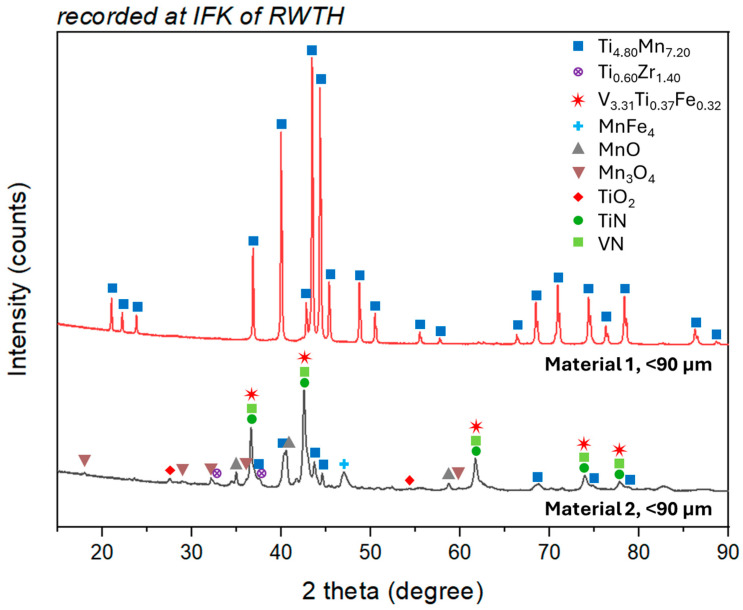
XRD of Material 1 and 2 (ground and sieved to <90 µm in both cases).

XRD analyses are used for phase analysis and only provide qualitative information, i.e., they only reveal which compounds are present and not how many. After phase determination, a Rietveld analysis can also be performed to add a quantitative dimension, but this becomes more complex as the number of phases increases and requires a high level of prior knowledge. For this reason, the analysis here is limited to the qualitative dimension. Figure 6 shows the diffraction lines (15 to 100° 2θ) for Material 1 and Material 2 which is ground under air and sieved to <90 μm. In the case of Material 1, only the main phase consisting of Mn and Ti is detected. In the case of Material 2, clear nitride peaks can be seen in addition to oxides such as TiO_2_ or Mn with varying degrees of oxidation. The nitride peaks can be attributed to titanium and vanadium nitride. Although significantly weakened, but still clearly identifiable, the original TiMn peaks and other metallic subphases consisting of V, Zr, and Fe are still present in Material 2. This metallic content can also be seen in the light microscope images, as described in the next chapter.

#### 2.1.3. Microscopic and SEM-EDX Analysis

**Figure 7:**
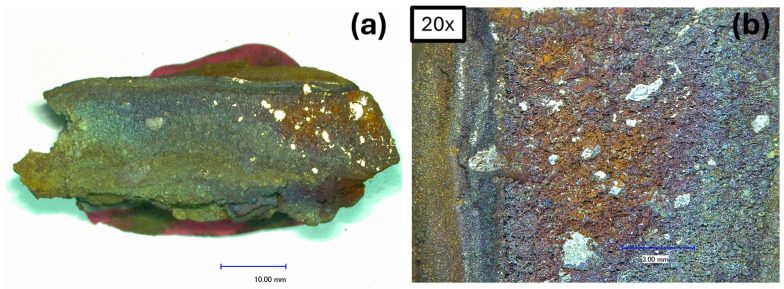
(**a**) Cross-section of representative Material 2 lump; (**b**) 20×.

A representative lump of Material 2 (like that shown in Figure 3) was cut in half and examined under an optical microscope. Figure 7a,b displays the cross-section of the cut sample. There are clearly visible metallic areas within a porous structure.

**Figure 8:**
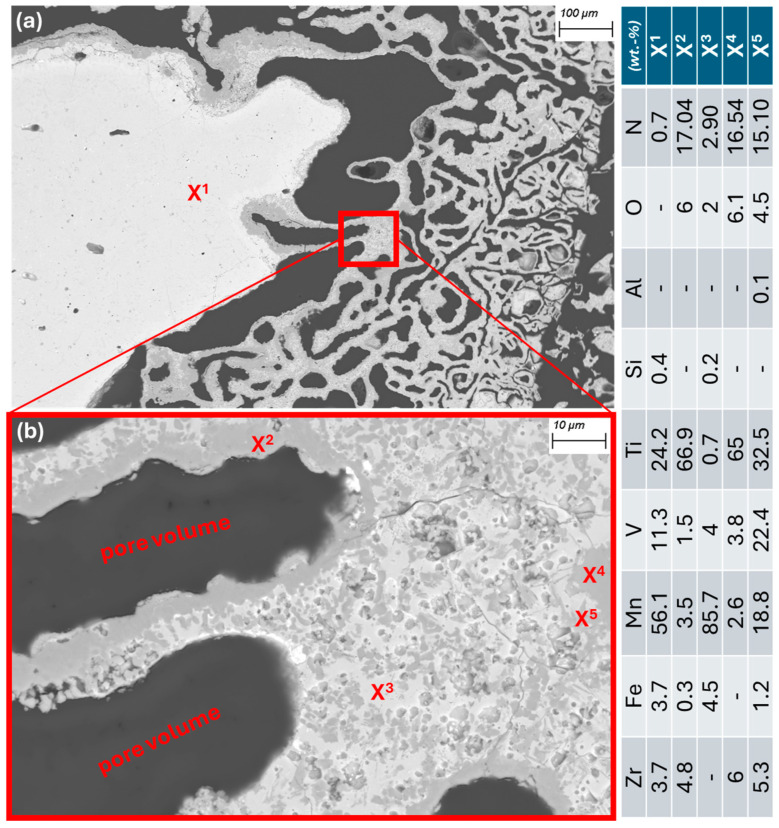
(**a**) Material 2 cross-section at 100× (BSE mode); (**b**) 1000× (BSE mode).

A closer look at the cut piece of Material 2 under the SEM-EDX provides more detailed insights. Figure 8a shows a metallic area and the representative surrounding structure as it appears throughout the sample. It is interspersed with pores (dark gray) of varying sizes. The matrix in which the pores are located is shown in Figure 8b. It can be seen that the interior of the matrix (X_3_) consists mainly of heavily Mn-enriched and slightly oxidized and nitrided areas. At the edges, i.e., the contact surfaces with the pores, there are thick layers of titanium oxynitrides (X_2_ and X_4_). Areas containing vanadium oxynitrides (X_5_) can also be found in their vicinity. The presence of larger areas containing Ti-N or Ti-V-N is consistent with the results of the XRD measurement mentioned above.

### 2.2. Determination of Basic Alloy Properties

**Figure 9:**
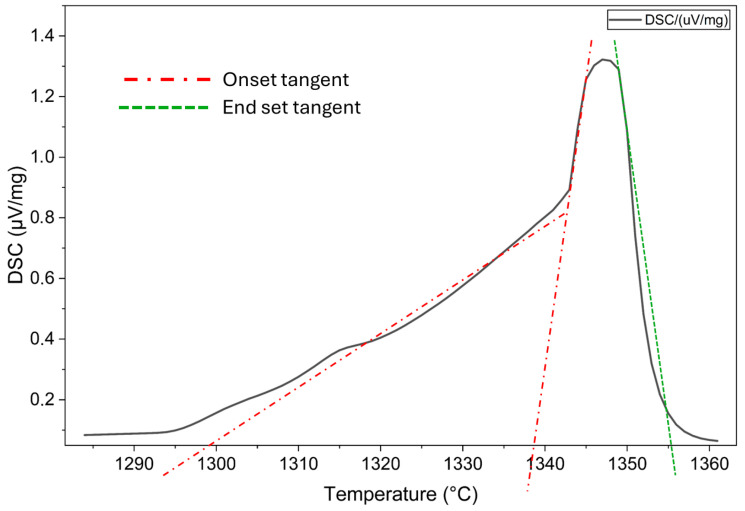
DSC measurement of Hydralloy C5 with onset and end set tangents plotted.

Melting of Hydralloy C5 is simulated in FactSage 8.3. Using the database SGTE, the software predicts a solidus temperature of 1147 °C and a liquidus temperature of 1249 °C. In addition to this a differential scanning calorimetry (DSC) measurement is performed by NETZSCH (NETZSCH DSC 404 F1 Pegasus^®^, Erich Netzsch B.V. & Co. Holding KG, Selb, Germany) with a heating rate of 20K/min. The onset and end set temperatures correspond to the intersection of the tangent at the voltage peak flank with the base line. While the onset temperature indicates the presence of the first liquid phase, the end set temperature indicates that most or all of the material is liquid [24]. Figure 9 shows the recorded DSC curve for Hydralloy C5. The normalized thermally induced voltage is plotted against the temperature. There is no typical bell curve, so depending on where the tangent is applied, the onset temperature is approximately 1300 or 1338 °C. However, the end set temperature is clearly approximately 1355 °C.

Besides the calculation in FactSage and DSC measurement, a separate test melt is also carried out. A thermocouple is placed in the center of the crucible, which in turn is located in an induction coil. The alloy is heated, everything is melted, and then the power is switched off. Without changing the heating power, a temperature plateau occurs at approx. 1298 °C. Once everything is liquid, the temperature continues to rise and the power is switched off. The temperature at the start of the cooling plateau is 1323 °C. This temperature corresponds fairly closely to the temperature at which the intermetallic TiMn_2_ C14-Laves phase precipitates in the binary Ti-Mn phase diagram. According to [25], this temperature is 1325 °C.

**Figure 10:**
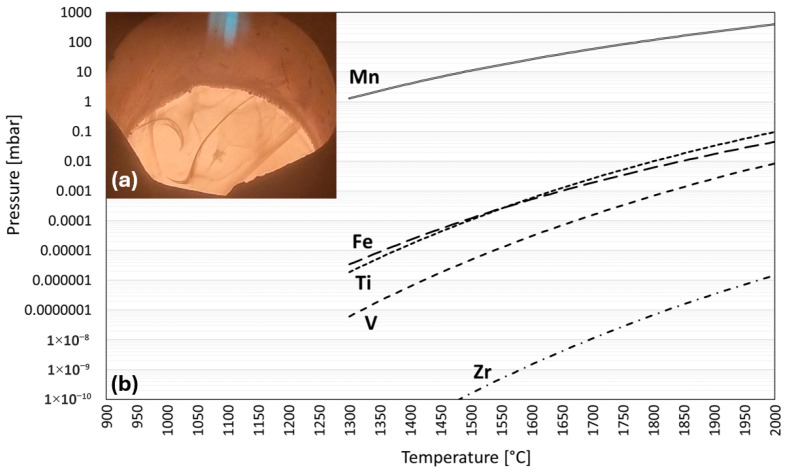
Manganese vaporizing during melting (**a**); equilibrium vapor pressures of the main components of Hydralloy C5 plotted against temperature (**b**).

The vapor pressure is a measure of the volatility of a component and is calculated by multiplying the (equilibrium) vapor pressure of the pure substance by the activity of the substance in the real solution. Both can be determined in FactSage. Figure 10b shows the equilibrium vapor pressure of the main alloying elements plotted against the temperature. The plot starts at 1300 °C, since, as previously determined, a liquid phase is only present approximately from this point onwards. It can be seen that manganese has a vapor pressure that is several orders of magnitude higher than that of the other components. At 1400 °C, the vapor pressure of manganese is approximately 4 mbar. The strong evaporation of an element begins as soon as the partial pressure of an element in the gas phase is lower than the vapor pressure of the same element in the liquid phase. This is exploited, for example, in the vacuum distillation of lead and zinc from brass [26]. However, even if the partial pressure does not fall below the vapor pressure, slight-to-moderate evaporation can still be expected. In Figure 10a, the smoke clouds of the evaporating manganese are clearly visible. Blacha et al. [27] demonstrate that this behavior of Mn is independent of the system. Their vapor pressure simulation of an OT4 alloy (94.29, 3.50, and 1.49 wt.% of Ti, Al, and Mn) shows that even at low Mn contents, its vapor pressure is orders of magnitude greater than that of other elements.

## 3. Materials and Methods

**Figure 11:**
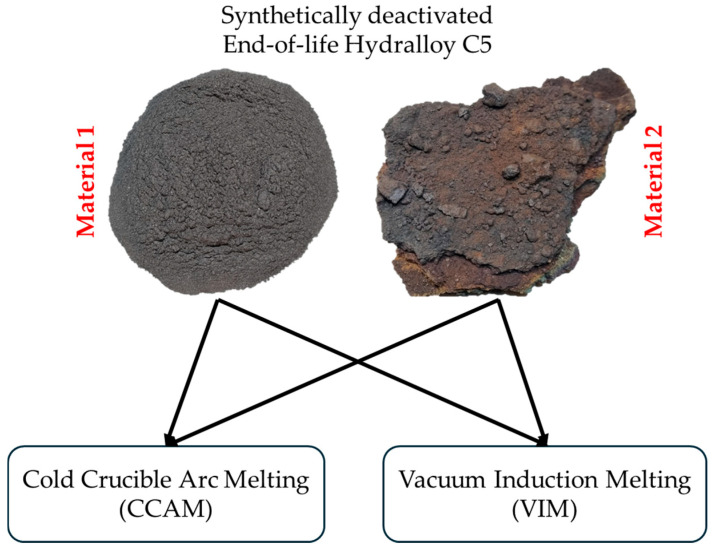
Preliminary trials conducted and melting aggregates used.

Since a vacuum induction melter (VIM) (VSG010, PVA TePla AG, Wettenberg, Germany) is used in the primary production of Hydralloy C5, we have decided to use it as a remelting unit. We have also chosen a cold crucible arc melter (CCAM) (AM200, Edmund Bühler GmbH, Bodelshausen, Germany) because of its simplicity and widespread use. Thus, as part of preliminary trials, we will charge Material 1 and Material 2 into the VIM and the CCAM as shown in Figure 11.

First, the experimental procedure for melting Material 1 and Material 2 in the CCAM is described. This is followed by the selection of suitable refractory materials for remelting Hydralloy C5 in the VIM. Finally, the procedure for the trial of Material 1 and Material 2 in the VIM is described.

### 3.1. Cold Crucible Arc Melting (CCAM)

Before the process is started, the chamber is evacuated to 5 × 10^−2^ mbar and flooded with argon to 700 mbar. This is repeated twice. Subsequently, an argon process pressure of 700 mbar is set. The initial current we start with is 50 A. It is gradually increased to 150 A in a time span of 30 s, which is then maintained for a further 1.5 min. This is followed by a flip of the molten material (under argon) and the melting is repeated. This procedure is the same for Material 1 and Material 2.

### 3.2. Vacuum Induction Melting (VIM)

#### 3.2.1. Selection of Suitable Refractories

To the best of the authors’ knowledge, there are no publications that investigate the interaction between liquid Hydralloy C5 and refractories. Only a few could be found, such as [2] or [28], which use similar alloy compositions (also TiMn2-based). These and a few other publications on melt–refractory interactions are listed in Table 2 as a reference for our crucible selection.

Li et al. [29] use an almost pure CaO crucible, but one that still contains 2 wt.% ZrO_2_. This is compared with graphite when melting TiFe_0.86_Mn_0.10_. While both materials result in similarly low O values in the melt (~0.08 wt.%), the C content is significantly higher when graphite is used (0.186 vs. 0.016 wt.%). Sticky TiC attachments to the crucible wall are also observed. One publication that remelts an alloy with a similar composition to that used in this work is [2]. It is merely stated that an Al_2_O_3_-based refractory is used. This leads to contamination of the cast hydrogen storage alloy with oxides, suboxides, and Ti-rich precipitates. Nonetheless the obtained alloys possess acceptable H_2_ capacities. The alloy used by [28] is also similar to Hydralloy C5. It is remelted in graphite and Y_2_O_3_-coated Al_2_O_3_-SiO_2_. Despite the approximately 0.05-inch-thick (1.27 mm) passivation layer, a reaction between crucible material and melt can be observed.

With a view to scaling up the recycling process in VIM at a later stage, very expensive (Y_2_O_3_) and difficult to handle (CaO) materials are initially excluded. Instead, Al_2_O_3_-based crucibles and ZrO_2_-containing refractories are selected.

#### 3.2.2. VIM Procedure and Material

Two different refractory materials are selected for testing in contact with Hydralloy C5. These are CaO-stabilized ZrO_2_ and titanium aluminate (TiO_2_-Al_2_O_3_). Titanium aluminate is an affordable and potentially thermochemically more stable material than pure Al_2_O_3_. And ZrO_2_ is a material that is in theory even more stable. In addition to the material, a slightly porous crucibles character is chosen, as densely sintered materials are much less resistant to the extreme temperature differences that occur during induction melting. Controlled, precisely regulated cooling is not possible with the VIM unit at hand, which means that thermal-shock-induced cracks would occur at this point at the latest. All crucibles used were purchased from GTS GmbH & Co. KG (Düsseldorf, Germany) and had the composition listed in Table 3.

**Figure 12:**
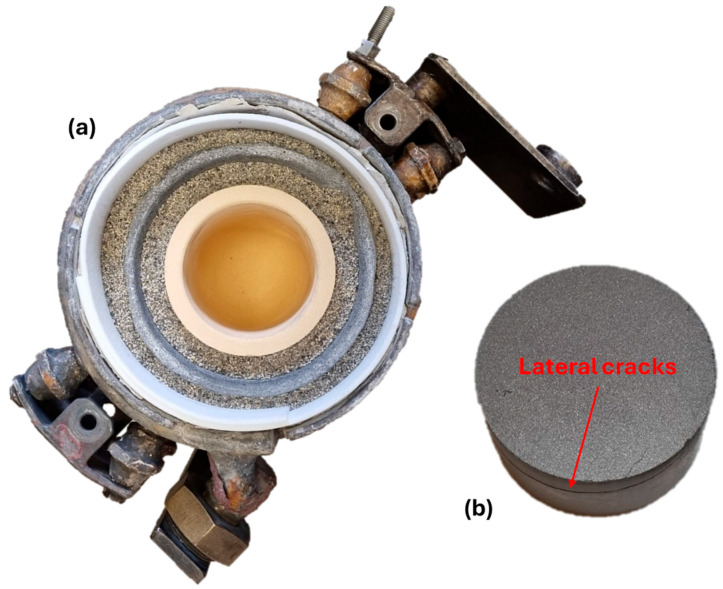
(**a**) Induction coil setup for refractory–melt interaction trials; (**b**) briquettes made from Material 1 seen in Figure 3.

A medium-frequency vacuum, melting, and casting cold wall furnace is used as the remelting unit. The operating frequency is between 8 and 10 kHz. The available induction coils in combination with the possible frequency are not sufficient to melt metallic powder material. The material must have a larger diameter in order to couple with the magnetic field. For this reason, the Hydralloy powder from Figure 3 is compressed (HTP40, HERZOG) to briquettes (40 mm diameter, 20–30 mm height) which, in combination with the use of a clay–graphite cylinder also placed in the coil around the crucible, couple sufficiently to be melted. The briquettes are generated at 60–150 kN for 20–30 s. Figure 12 shows the setup within the coil as described (a) and the briquetted Material 1 powder (b). During uniaxial briquetting in the cylindrical press die, lateral cracks occur regardless of the variation of parameters such as press force and duration. These cracks lead to instability of the briquettes, but do not significantly impair the coupling behavior.

Based on the initial findings regarding Hydralloy C5’s basic properties displayed in one of the previous chapters, we conclude that the Material 1 trials should take place at temperatures of approx. 1400–1500 °C and a rather high pressure. The process pressure is set at 700 mbar. A further increase is not possible due to furnace design reasons. These parameters guarantee that a completely liquid phase can be expected and that the evaporation of manganese is kept within limits.

The trial procedure is as follows: The crucible is filled to the brim with Material 1 briquettes. After the lid is closed, the furnace is evacuated to approx. 5 × 10^−2^ mbar and then flooded with argon to 700 mbar. This is repeated twice. The furnace is heated in a vacuum to a temperature of 1000 °C until the process pressure of 700 mbar is applied. Once everything has melted, additional virgin briquettes are added via the furnace’s lock system using an excavator until a total mass of ~2.3 kg of Material 1 is melted in the crucible. This entire process takes approximately 300 min. After that, the holding time of 30 min begins. After this period has expired, the melt is cast into a water-cooled copper mold.

After casting, the system cools down under argon. The furnace lid is only opened once room temperature has been reached. The crucibles are then removed from the induction coil and cut lengthwise down the middle to remove a piece for SEM-EDX analysis.

A modified setup must be selected for the remelting trial of Material 2 in the VIM. Due to the relatively low metal content, the pieces (as shown in Figure 3) do not couple well to the induction field. Grinding and briquetting would not remedy this, as the non-metallic (and therefore non-coupling) content remains the same. In fact, grinding Material 2 would tend to reduce the chances of recovering the metallic content due to dispersion of the metallic parts. For this reason, a graphite crucible is coated several times with Y_2_O_3_ and approx. 800 g of Material 2 is charged. Once the process temperature of 1400–1500 °C has been reached, it is maintained for 30 min. The furnace is then switched off, and the system cools down under argon. Subsequently, it is examined whether a metal phase has formed and settled at the bottom.

## 4. Results and Discussion of Remelting Trials

### 4.1. Cold Crucible Arc Melting (CCAM)

#### 4.1.1. Material 1 (Low Contamination)

**Figure 13:**
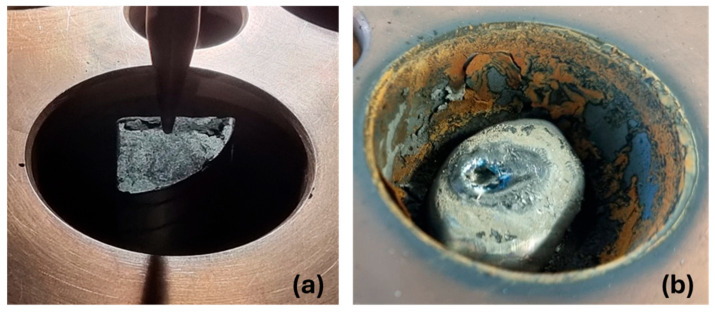
Cut briquette of Material 1 before remelting (**a**); Material 1 after remelting (**b**).

Figure 13 shows that at the end of the melting process, a completely molten metal phase is present. Of the 22.57 g initially charged as a briquette, 21.78 g remain after one flip of the metal (i.e., two remeltings), which corresponds to a weight loss of approximately 3.5%. The weight loss is due to evaporated manganese. This mainly condenses in the water-cooled copper pot (as shown in Figure 13b). The ingot melted from Material 1 is just as brittle as the virgin Hydralloy C5. This indicates that no significant phase change has taken place and that it is still intermetallic.

#### 4.1.2. Material 2 (High Contamination)

**Figure 14:**
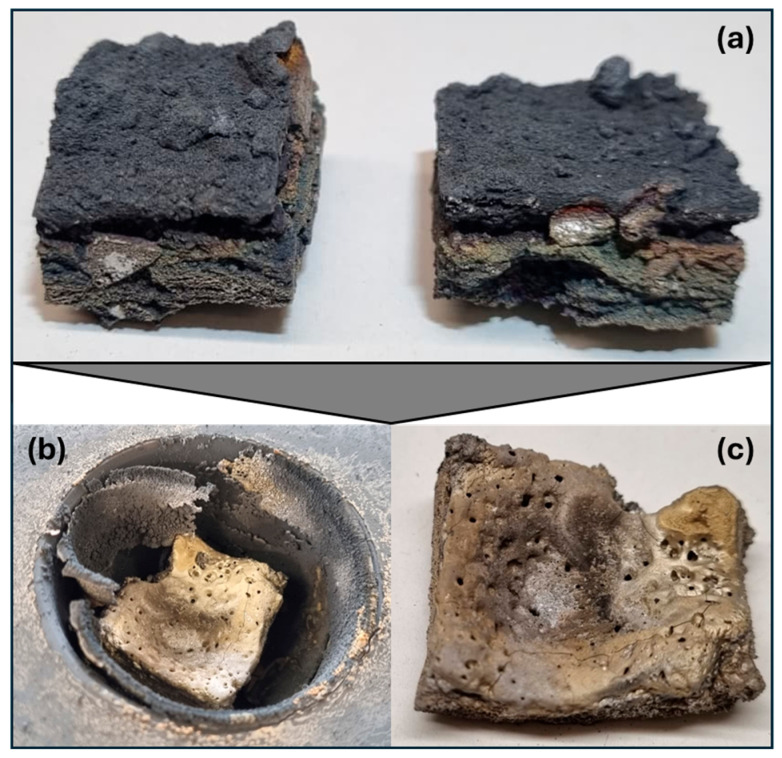
(**a**) Synthetically deactivated Hydralloy C5 used; (**b**) Partially melted piece in CCAM copper mold; (**c**) Close-up of the partially melted piece.

The melting process had to be stopped after about one minute because visibility became too poor due to heavy smoke. Figure 14c shows that the height of the piece used has been greatly reduced. A separate phase from the metallic residues, as shown in Figure 14a, cannot be found. It can be assumed that they have evaporated. The evaporated material has settled on the entire water-cooled copper plate but has mainly condensed in the recess (see Figure 14b).

**Figure 15:**
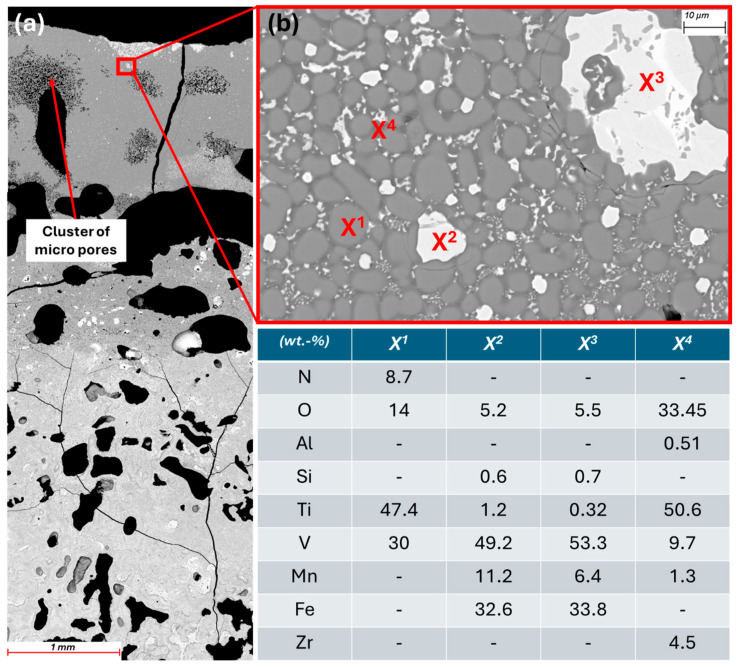
(**a**) Complete cross-section of the synthetic deactivated Hydralloy C5 melted in CCAM from Figure 14c at 50× magnification (BSE mode); (**b**) 1000× magnification of the molten upper area (BSE mode).

SEM images were taken at 50× magnification across the entire cross-section of the piece from Figure 14c. The images were combined to form Figure 15a, so that the entire cross-section is shown in one image. It can be seen that only the upper quarter is molten, while the original structure with pores is still present below. The reason for this is probably the poor thermal conductivity of the oxide and nitride components. In the molten area (see Figure 15b), a structure has formed in which the matrix consists of globulitic Ti-V-oxinitride (X_1_) coated with titanium oxide (X_4_). Islands of Fe-V oxide (X_2_, X_3_) is present in this matrix. The manganese-rich phase X_3_ in Figure 8b no longer exists. In general, the manganese content of the phases present in the molten area is very low, which is due to its evaporation. It was expected that some of the manganese would evaporate, especially since the vapor pressure according to the FactSage calculation in Figure 10b is already 400 mbar at 2000 °C. However, such a high degree of volatilization was not anticipated. This, coupled with the fact that no metallic phase can be identified after melting, leads to the conclusion that simple remelting is not possible.

### 4.2. Vacuum Induction Melting (VIM)

#### 4.2.1. Material 1 (Low Contamination)

**Material deposition**

**Figure 16:**
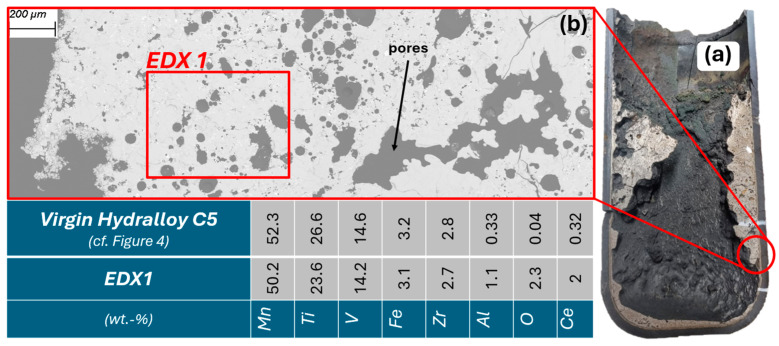
(**a**) Titanium aluminate crucible cut lengthwise; (**b**) 45× magnification of crucible wall deposits with EDX1 being a field measurement (BSE mode).

As planned, the two interaction trials are cast at approximately 1450 °C. For CaO-stabilized ZrO_2_ and titanium aluminate, the exact casting temperatures are 1428 and 1490 °C. However, it is only after casting that it becomes apparent that thick deposits have formed on the inside of the crucible wall. At first glance, they look like slag but cutting them open (see Figure 16a) reveals that they are metallic. Figure 16b shows the cross-section of the deposits under the SEM. A porous structure can be seen with a similar composition (EDX1 = area measurement) to that of virgin Hydralloy C5. The main alloying elements show only slight deviations. These are probably due to the use of a different measurement method (EDX instead of ICP-OES). The deviations with regard to Al, O, and Ce require further explanation. The Al may come either from the Al_2_O_3_ coating used or from interaction with the crucible material (titanium aluminate). No Al or Al-mixed oxides can be identified in EDX point measurements. Instead, Ti, Zr, Zr-Ce, and Ce-La oxides are found, which indicates the reduction of aluminum oxide by these elements.

**Figure 17:**
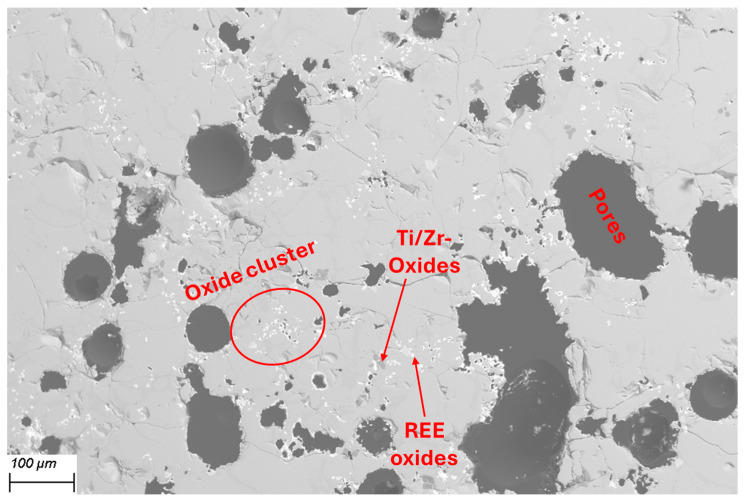
100× mag. of the EDX1 section from Figure 16b with dispersed oxides (BSE mode).

The deposits on the crucible wall occur regardless of the refractory material used. They are always interspersed with the oxides mentioned above. While Ti and Zr oxides increase toward the crucible wall (interactions with refractory materials are discussed in detail below), rare earth element (REE) oxides (Ce, La) are distributed across the entire deposit area. Figure 17 depicts the EDX1 section from Figure 16b at 100× magnification, where the dispersed oxides can be seen as white-gray dots.

It is questionable whether the deposits would be dissolved by a longer holding time, since the holding time in the trials carried out was already 30 min with temperatures always around 1450 °C up to 1500 °C. It is possible that parts of the magnetic field are intercepted by the graphite cylinder used, resulting in significantly less inductive stirring. The fact that graphite couples inductively very well and thus has a shielding effect is described, for example, in [30]. Besides the lingering of the wall deposits, their actual formation could be attributed to the melting process and the material used. Since Material 1 was generated by grinding under air, it can be assumed that there is a more or less thin oxide skin around each individual metal particle. The smaller the particles, the more reacted surface area there might be. When the briquettes were molten down, a metal-oxide slurry may have formed in some cases, which then led to the formation of the inclusions as seen in Figure 17. Although the oxygen content of Material 1 is only around 0.15 wt.%, patent [1] states that increased oxygen content can be expected to result in (what they call) dross formation. The patent for the primary production of Hydralloy C5 cites five examples for melting the alloy. These are listed in Table 4. The first four melts are carried out in two stages, i.e., the MnFeV master alloy is melted in the first step. It has a virtually constant, uniformly low O content. The only variation is in the amount of cerium mischmetal added (between 0.6 and 2 wt.%). In the fifth example, melting is carried out in one single stage, and all the required materials are alloyed in one step. It is stated that “due to the high oxygen content of the materials used, in particular FeV80 [0.9 wt.% O], considerable dross formation occurred during deoxidation.” It is further stated that the dross adhered to the crucible and led to yield losses of ~10 wt.%. The yield losses in the VIM melts carried out in this work amount to ~45 wt.%. However, this significant amount is most likely due to the fact that in the case of the patent melt (Ex. 5), there is a significantly larger volume in relation to the crucible surface area (cf. our feed amount ≈ 2.5 kg, their feed amount ≈ 15.5 kg).

**Refractory–melt interactions**

**Figure 18:**
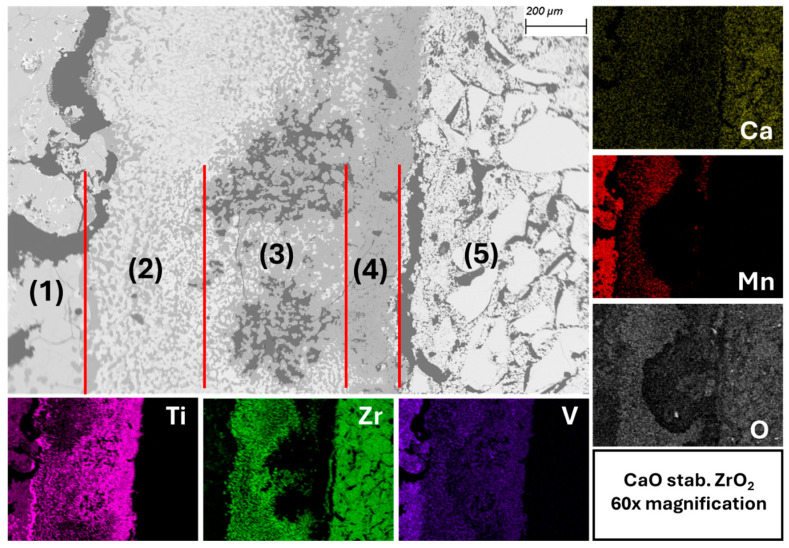
Multi-layer contact zone between melt and CaO-stabilized ZrO_2_ (BSE mode) with EDX mapping.

The multi-layer contact zone between the melt and the CaO-stab. ZrO_2_ crucible wall is depicted in Figure 18. The individual layers are numbered and marked with red lines to distinguish them from one another. The EDX mapping shows, based on the color intensity, that there is an increased amount of Ti and a severe deficiency of Mn and Zr directly at the crucible wall. Fe exhibits the same distribution behavior as Mn. While Ce is evenly distributed in the transition zone, La always appears together with Ti on the mapping. However, distinct Ti-La phases could not be identified in EDX point measurements. These observations are independent of the crucible material.

Starting on the left is the melt (1). This is followed by zone (2), which contains a mixed structure of substoichiometric ZrO_{1.85 (ratio between O and the respective element (in this case Zr))} and titanium enriched with oxygen. The oxygen content of approx. 33 at. % would still be within the solubility range of α-titanium according to [31]. However, this solubility is only present at high temperatures. Below approx. 600 °C, oxides with a high titanium content form. Ref. [32] (p. 559) is a publication that also reports on the formation of α-Ti(O) and oxygen-deficient zirconium oxides upon contact between molten titanium and ZrO_2_. Zone (3) consists mainly of strongly substoichiometric TiO_{0.67} with a few light-gray areas in between, which represent ZrO_{1.85}, and branched prone clusters. Zone (4) directly adjacent to the crucible wall consists almost exclusively of substoichiometric titanium oxide (TiO_{0.88}) and dioxide (TiO_2__{1.75}). Slightly substoichiometric ZrO_{1.95} can be identified on the crucible wall. ZrO_2_ is present in the crucible itself (5). No infiltration of the crucible can be seen on the SEM-EDX images.

What this observation shows, on the one hand, is that the transition zone forming between the melt and the crucible wall is strongly dominated by Zr and Ti. While increasingly oxygen-rich titanium phases occur towards the crucible wall, the opposite effect is observed for zirconium-containing phases. A similar effect is observed [33] when melting pure titanium in fused CaZrO_3_. They also report diffusion of Zr and O into the melt. Unlike in this study, however, infiltration of the crucible material occurs. While the corrosion of CaZrO_3_ in contact with pure titanium is determined by infiltration and Zr + O diffusion, our test results suggest that in the case of Hydralloy C5 and CaO-stab. ZrO_2_, it is only diffusion. Diffusion processes of varying speeds may also explain the locally occurring pore structures as seen in (3) in Figure 18. It is concluded in [33] that Ti can be melted in CaZrO_3_ if refractory infiltration and thus corrosion is avoided or limited. This infiltration of the Ti melt into the CaZrO_3_ crucible was observed. In the case of the Hydralloy C5 melt, no infiltration into the CaO-stab. ZrO_2_ crucible was observed.

**Figure 19:**
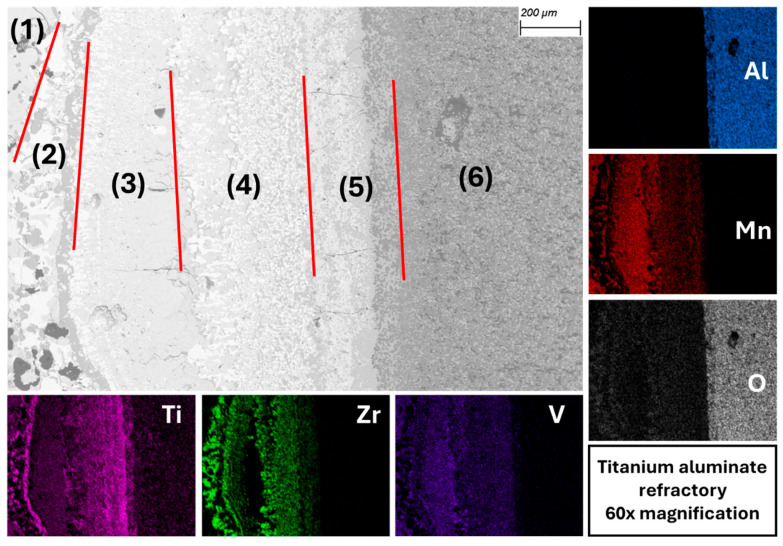
Multi-layer contact zone between melt and titanium aluminate (BSE mode) with EDX mapping.

When titanium aluminate comes into contact with the Hydralloy C5 melt, as in the case of CaO-stabilized ZrO_2_, a multi-layer contact zone forms between the melt and the crucible wall. As before, the individual layers are numbered and marked by red lines separating them from one another. Figure 19 illustrates this, and that the contact zone is approximately one millimeter thick. The dominant elements in the transition layer are Ti and Zr. It is noteworthy that Zr is very strongly represented there despite its low alloy content of approximately 3 wt.%, as can be seen from the color intensity. Only zone (3) represents an area consisting largely of alloy melt with virtually no zirconium in it. The elements Mn and V occur throughout the transition zone but play a subordinate role. They are contained in alloy melt areas located between the oxides.

Starting with the melt, this represents zone (1). Here, the first Ti and Zr oxides (ZrO_{1.28}, ZrO_{2}, and TiO_{0.65}) can already be found, as well as an alloy phase that is heavily depleted in Ti and Zr. Zone (2) represents an area consisting of Zr oxide (ZrO_{1.75} and ZrO_{1.82}), a melt heavily depleted in Zr, and a pronounced Ti phase with approx. 14 wt.% oxygen. The latter is probably high-titanium-containing oxides, since α-Ti is not stable at RT with such a high oxygen content (as already described above). This phase forms a 10–20 μm thick layer (dark gray) that runs vertically through the image. It can also be seen with the other refractory material in Figure 18. In (3), ZrO_{1.8} is present in a matrix consisting of a strongly Zr-depleted alloy melt. Zone (4), on the other hand, consists of ZrO_{1.78} and TiO_{0.7} embedded in a Ti- and Zr-depleted and Al-enriched alloy melt. The aluminum content is between 1.5 and 3 wt.%. While (6) shows the untouched refractory material, (5) depicts the area of the crucible wall that has been infiltrated. In the infiltrated area, the Al_2_O_3_ density has decreased in favor of titanium oxides such as TiO_{0.87}, TiO_{0.9}, and TiO_{1.05}. Against this background and given the increased proportion of Al in the surrounding alloy melt, it is clear that a reduction of aluminum oxide by Zr and/or Ti has taken place. The extent to which TiO_2_ has been reduced from the refractory material is unclear. However, it is generally known that substoichiometric Ti oxides can form through contact between TiO_2_ and liquid Ti [34].

#### 4.2.2. Material 2 (High Contamination)

**Figure 20:**
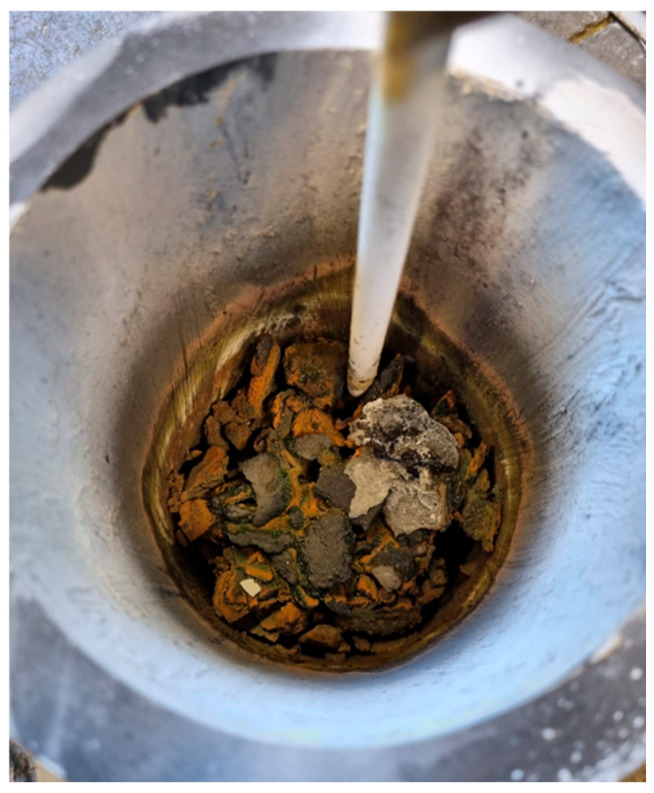
Material 2 after holding between 1400 and 1500 °C for approx. 30 min in the VIM.

The result of the remelting test of Material 2 in the VIM can be seen in Figure 20. Although a temperature between 1400 and 1500 °C was maintained for approx. 30 min, no separate metal phase can be identified. It is very likely that the metallic areas partially present in Material 2 are too much enclosed by non-metallic components (see Figure 7), preventing separation from taking place.

## 5. Summary

The basic alloy properties in the molten state were studied on virgin Hydralloy C5. From thermochemical simulations and practical experiments, it can be derived that the range between the first liquid fraction and a completely liquid phase lays approx. between 1300 °C and 1350 °C. The vapor pressure of Mn in Hydralloy C5 is several orders of magnitude higher than that of all other alloying elements. In practice the evaporation of Mn begins as soon as the first molten fraction is present. All other alloying elements remain in the molten phase.

The hydrogen-cycled and suddenly air-exposed Hydralloy C5 (Material 2) exhibits high O and N contents (~1 and ~8 wt.%) with areas of unreacted metal. The high proportion of nitrides (TiN, VN) in particular poses a problem, as they are very stable thermally, mechanically, and chemically. Separating the remaining metallic areas from the non-metallic components cannot be achieved by simple melting in VIM or CCAM. It is concluded that there is no way around developing a significantly gentler, i.e., less reactive, method of opening the real storage cylinders in order to receive less O- and/or N-contaminated Hydralloy C5. Only with significantly less contaminated alloy material is it worthwhile to undertake further metallurgical melting efforts. This is demonstrated by the remelting trials conducted on Hydralloy C5 material, which was only slightly contaminated (Material 1). The O and N contents in the powder generated by grinding in air are only ~0.15 and ~0.04 wt.%, respectively. At these contamination levels, the alloy powder can already be safely handled in ambient air and be melted in the VIM as well as CCAM. The key difference between the two methods for synthesizing end-of-life Hydralloy C5 powder with two different levels of contamination is the availability of a reactive atmosphere. A sudden and uncontrolled exposure to ambient air triggers a continuous reaction mechanism (flaring). The high temperatures generated in this process are very likely what enables the excessive formation of nitrides. In the grinding vessel, however, where the same ambient atmosphere prevails but only to a limited extent, no formation of nitrides takes place (as evidenced by the unchanged N content compared to virgin Hydralloy C5). This fact demonstrates that, when real storage containers are opened in the future, the medium used for deactivation should be introduced in carefully controlled doses.

When remelting the slightly contaminated Hydralloy C5 (Material 1) in the VIM, it can be observed that thick deposits form on the inner wall of the crucible, regardless of the refractory material selected. They significantly reduce the melt yield and do not dissolve even after prolonged holding times. Against this background, consideration must be given to how to deal with these thick deposits in the VIM. It may be necessary to use a (deoxidizing) flux to remove the non-metallic inclusions from the metal phase. This is common practice for binding non-metallic components that would otherwise adhere to the inner walls of the crucible during induction melting, as shown in [35]. The crux of the matter will be finding a flux that is suitable for the alloy and refractory system at the same time.

The SEM-EDX images of the contact surfaces show that in case of titanium aluminate, an infiltration layer can be seen. This layer is homogeneous and uniformly thick. In this layer, the Al_2_O_3_ content decreases significantly in favor of TiO_2_. No infiltration has taken place in CaO-stabilized ZrO_2_, but it can be seen that Ti has interacted with ZrO_2_ and formed a layer approximately 150–200 µm thick on the crucible wall surface. In both refractory materials, the transition layer is dominated by Ti and Zr. This is particularly remarkable since Zr only accounts for approx. 3 wt.-% of the alloy. Due to their high oxygen affinity, Ti and Zr are selectively oxidized by reacting with Al_2_O_3_, forming substoichiometric Zr and Ti oxides. The multi-layer transition zones are most likely formed by diffusion processes. With regard to the REEs Ce and La, which also have a high affinity for oxygen, while Ce is distributed evenly across the transition layer, La tends to concentrate where Ti is also present. Both elements are present exclusively as oxides or mixed oxides, indicating that, despite their very low content of <0.4% by weight, they play a role in reduction reactions.

## 6. Conclusions and Outlook

Neither VIM nor CCAM was able to extract a molten metal phase from the heavily contaminated end-of-life Hydralloy C5 material. Consequently, an extraction method must be developed that results in significantly lower contamination levels.

With regard to safe handling of the powdered Hydralloy C5 in ambient air, O contents of 0.1–0.2 wt.% already seem to be sufficient for passivation. However, these contents also already appear to be sufficient to form crucible wall deposits in the VIM due to the formation of oxide–metal agglomerates, which lead to significant losses in melting yield. Losses also occur when melting the slightly contaminated end-of-life Hydralloy C5 material in the CCAM. These amount to approx. 3.5 wt.% and are due to the evaporation of manganese.

With regard to melt/refractory interactions, it can be concluded that multiphase refractory mixtures with Al_2_O_3_ should be avoided due to reduction by the Ti and Zr contained in Hydralloy C5. CaO-stabilized ZrO_2_, on the other hand, withstood several hours of contact with molten Hydralloy C5 well.

Based on the insights generated from this publication, the following aspects are being considered for the future:

- Once the method for gently opening the real storage containers has been established, we will know the exact level of O and N contamination that needs to be adjusted for during recycling. This real contaminated end-of-life Hydralloy C5 can then be compared with the simulated materials from this publication. This applies not only with regard to chemical composition, morphology or particle size distribution, but particularly with regard to melting behavior.
- We will remelt the real end-of-life Hydralloy C5 with the realistic contamination level in the VIM and CCAM, if necessary with the gradual addition of the deoxidizer cerium mixed metal, which is also used in primary alloy production. With regard to the separation of non-metallic inclusions and thus to avoid crucible wall deposits, we will conduct several VIM trials using fluxes. The remelting tests to be carried out in the CCAM and VIM in the future will be accompanied by H_2_ capacity measurements.
- Further refractory–melt interaction tests will take place in the VIM, this time using only virgin 2–10 mm Hydralloy C5. Refractory materials such as magnesium aluminate spinel and refractories coated with Y_2_O_3_ and fired will be used.

## Figures and Tables

**Figure 2:**
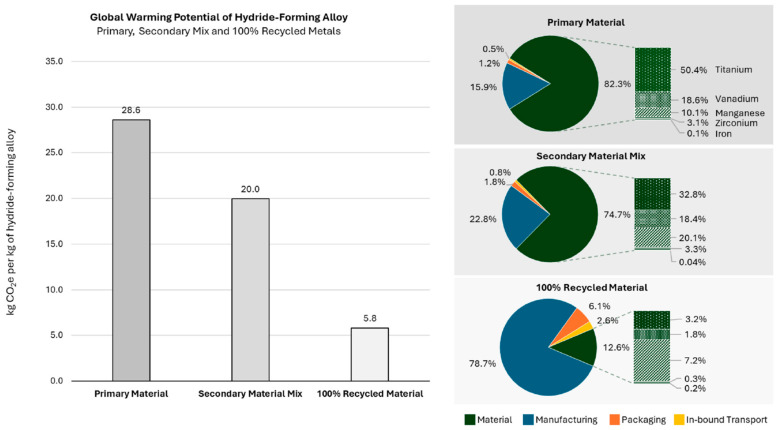
Global warming potential (GWP) results for primary, mixed and secondary Hydralloy C5 production scenario (**left**); proportional origins of CO_2_ emissions with respect to raw material to factory gate (**right**) [8].

**Table 1:** Evaluation of the materials used for Hydralloy C5 according to various criteria.

Input Materials for Hydralloy C5	EU Criticality (2023) [22]	EU Import Reliance (Primary) [22,23]	EU Import Reliance (Refined) [22,23]
Iron deriv. from [3,17]	Iron (not CRM) Coke (CRM)	77% (Ore) 66% (Coking coal)	5% (Pig iron, DRI, granules and powders, crude steel) 0% (Coke, tar, benzole, ammonia, coke oven gas)
Aluminum [17]	Aluminum/Bauxite (CRM, SRM)	89% (Bauxite)	58% (Refined alumina, unwrought aluminum)
Vanadium oxide [17]	Vanadium (CRM)	100% (Ore, concentrate, crude oil, slag from steelmaking)	100% (Vanadium oxide, FeV, other chemical compounds)
Electrolytic manganese [1,2,8]	Manganese (CRM, SRM)	96% (Ore)	61% (FeMn, FeSiMn, manganese)
Titanium (Ti-Sponge) [21]	Titanium (CRM, SRM)	100% (Ore, concentrate)	18% (Ti-Sponge) 100% (Titanium)
Zirconium [23] (Zr-Sponge) [2,7]	Zirconium (no CRM)	83% (Zircon sand)	27% (Zirconium)
Cerium mischmetal [21]	Cerium (CRM, SRM) Lanthanum (CRM)	100% (Cerium concentrates) 100% (Lanthanum concentrates)	

**Table 2:** The melting literature on hydrogen storage alloys and refractories.

Alloy	Form	Crucible	Temp. [°C]	Hold. Time [min]	Main Take Away	Ref.
TiFe_0.86_Mn_0.10_	elemental	CaO-ZrO_2_ (98-2 wt.%) Graphite	1500–1600	5–10	O content same for both, while C heavily increased in graphite case which went along with sticky TiC attachments	[29]
Ti_0.85_Zr_0.15_Mn_1.22_Ni_0.22_Cr_0.2_V_0.3_Fe_0.06_	elemental	Graphite Y_2_O_3_-lined Al_2_O_3_-SiO_2_	1600–1800	1	Despite the 0.05-inch spray-painted Y_2_O_3_ layer, the melt in both crucibles was partially contaminated	[28]
Ti_1-x_Zr_x_Cr_y1_Mn_y2_Ni_y3_ Fe_y4_V_y5_	elemental	Al_2_O_3_-based	1600–1800	1–5	Contamination of ingots (oxides, sub-oxides and Ti-rich precipitates) from melt–crucible interaction but still acceptable H_2_ capa.	[2]

**Table 3:** Composition of refractories used for melt–crucible interaction.

(In wt.-%)	Al_2_O_3_	CaO	ZrO_2_	SiO_2_	Fe_2_O_3_	TiO_2_	Porosity [%]	Volume [mL]
CaO-stabilized ZrO_2_	-	4	94	0.4	-	-	20.5	600
Titanium aluminate	>70	-	-	-	-	<30	18	600

**Table 4:** Comparison of all five Hydralloy C5 production melts from the patent [1].

(In wt.%)	Ex. 1	Ex. 2	Ex. 3	Ex. 4	Ex. 5
Process steps	Two	Two	Two	Two	One
CerMM input	2.0	1.0	0.6	2.0	3.0
O in MnFeV	0.040	0.023	0.040	0.023	-
O in Hydralloy C5	0.040	0.050	0.040	0.030	0.260
CerMM in Hydralloy C5	0.55	0.16	0.13	0.85	0.51
H_2_ capacity	2.04	1.96	2.01	2.05	1.85

CerMM = cerium mischmetal.

## Data Availability

The original contributions presented in this study are included in the article. Further inquiries can be directed to the corresponding author.

## Abbreviations

The following abbreviations are used in this manuscript:

VIM	Vacuum Induction Melting
PIM	Pressure Induction Melting
CCAM	Cold Crucible Arc Melting
PESR	Pressure Electro Slag Remelting
ESR	Electro Slag Remelting
HSA	Hydrogen Storage Alloy
ICP-OES	Inductively Coupled Plasma Optical Emission Spectroscopy
SEM-EDX	Scanning Electron Microscopy with Energy Dispersive X-ray spectroscopy
NiMHB	Nickel Metal Hydride Battery
XRD	X-ray Diffraction
LCA	Life Cycle Assessment
CED	Cumulative Energy Demand
GWP	Global Warming Potential
DSC	Differential Scanning Calorimetry
CRM	Critical Raw Materials
SRM	Strategic Raw Materials
BCC	Body-Centered Cubic
BSE	Backscattered Electrons
